# The BioStudies database

**DOI:** 10.15252/msb.20156658

**Published:** 2015-12-28

**Authors:** Jo McEntyre, Ugis Sarkans, Alvis Brazma

**Affiliations:** ^1^ European Molecular Biology LaboratoryEuropean Bioinformatics Institute (EMBL‐EBI)Wellcome Trust Genome CampusHinxtonCambridgeUK

**Keywords:** Methods & Resources

## Abstract

The BioStudies database is a new EMBL‐EBI resource that holds descriptions of biological studies, links to supporting data in other databases, and archives data files that do not fit in existing public structured archives.

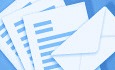

## Introduction

Making data and materials used in scientific research available to other researchers is the basis of scientific progress, as reconfirmed in the report of American Academy of Sciences more than a decade ago (National Research Council (US) Committee on Responsibilities of Authorship in the Biological Sciences, 2003) and more recently in the Royal Society Science as an Open Enterprise report (Royal Society, 2012). Abiding by this principle is not only necessary for ensuring reproducible science and maximizing the utility of public funding, but arguably even more importantly, to allow scientists to build on the previous work of their colleagues.

Since the inception of protein crystallographic structure and nucleotide sequence databases, it has been common practice to deposit a number of specific types of data in domain‐specific, community‐endorsed archival database and then cite the accession number in the accompanying research article (Kahn & Hazledine, 1988; International Union of Crystallography Commission on Biological Macromolecules, 1989). The advantages of this are twofold: first, data that underpin scientific assertions in the article are accessible, and second, the data are computationally reusable because the same data formats and standards are applied across the entire database.

However, in the age of data‐driven science, continuing to meet these aspirations comprehensively can be non‐trivial. The variety and volume of biological research data being produced is growing—nucleotide sequencing data alone are measurable in petabytes (and conceivably will soon be exabytes). There is a growing number of transcriptomic, proteomic and metabolomic technologies, as well as increasing amounts of research data from consented human individuals, adding further complexity. These and many other data types are supported by the public repositories exemplified by the protein crystallographic structure and nucleotide sequence databases, which work with journals to ensure that data are deposited in the most appropriate place. In addition, the availability of general‐purpose repositories such as Dryad, Zenodo or Figshare is helping to making unstructured “supplemental data” citable and more discoverable.

This situation is not only challenging for data resources—it affects everyone involved in reporting and publishing scientific outputs, most notably researchers and journals. For researchers, it is sometimes hard to know where to preferentially deposit multiple different data types and what to do with the supplemental data for an article, if anything. Journals (or rather, their editors) are increasingly required to have an encyclopaedic knowledge of data types and resources and frequently find themselves in a position where they need to advise researchers on where to deposit data on article submission and help enforce public data mandates. While several leading journals are injecting rigour into the availability of data behind the articles (Bloom *et al*, 2014; Data Citation Synthesis Group, 2014; Editorial Nature Cell Biology, 2014), it is fair to say that there is significant heterogeneity in the way that journals address the stewardship of data that support articles. Many challenges remain to be addressed, not least in the case of molecular imaging datasets, which can reach terabytes in size, for which new formats are frequently invented and for which no recognized solution as yet exists.

Equally importantly, finding or discovering all the data associated with a study and understanding the cross‐dependencies between data that may reside in different locations and formats can be tricky. The primary goal of the BioStudies Database therefore is to collect all the data from a study in a single record. In a simple case, a BioStudies record is a data‐centric reflection of an article, clustering links to data and files that underlie a study into a single citable unit.

## Use cases for the BioStudies database

As scientific experiments become increasingly data driven, and produce and combine data of a variety of types and scales, not all the outputs from a given study are addressed effectively via the narrative of an article. Frequently, the data behind a study go far beyond what is desirable to discuss in a typical paper and what gets cited are the highlights and not the full complement of data. A BioStudies record allows all pertinent data to be aggregated in one place and cited as a whole from the paper or elsewhere, removing the requirement to dig around in text to find links, leaving the article to focus on the story. In the case that a study produces more than one paper, which is not so unusual these days, a BioStudies record provides a single point of reference, creating a new way to link articles via the full complement of underlying data. As BioStudies can take submissions independently of article publishing workflows, there is also the opportunity to aggregate data for studies that may never be published in the conventional manner, for example, for negative findings or helpful but minor additional evidence to an existing study.

One of the great strengths of a research article is that it is a permanent object on which to build further. Ideally, data are deposited prior to the articles being published so can be cited in the narrative. Prior to article publication, BioStudies could be considered storage for all the underlying data links and files, and equally, post‐publication as a venue to add more supporting data in response to feedback once the article has been published. Once a BioStudy is linked from an article, any data added post‐publication, or additional descriptive information supplied, remain findable from the article. The BioStudy in this case then represents the full and up‐to‐date picture of supporting data.

Finally, positioning BioStudies in the context of other life science data resources such as those hosted by the EMBL‐EBI will provide opportunities for better data interoperability and data stewardship in the future. It will make it much easier to, for example, support journal editors on best practices in data deposition by providing insurance that data accidentally misplaced in unstructured data files can end up in the appropriate repository. It will also enable linking of BioStudy records from other life science data resources, as is already done across structured data resources. As new data types materialize in BioStudies depositions, this resource may provide an early warning system for emerging requirements for the development of new structured data repositories.

## The BioStudies database

The BioStudies database holds descriptions of biological studies with links to the underlying data in EMBL‐EBI databases or elsewhere. This defines the scope of BioStudies: if the study is supported by data available at EMBL‐EBI or associated to full text articles in Europe PMC, then it can be included in BioStudies. When necessary, BioStudies can also host data that do not fit in the existing structured archives.

We define a study as a biological experiment or set of experiments that are usually, but not exclusively, linked to an article. Studies may be of different scales or granularities; for example, the 1,000 Genomes Project can be considered one large‐scale study, but may include or be linked to many other (smaller) studies; studies may therefore have a simple hierarchical structure. From the implementation perspective, BioStudies can be seen as a system for managing data files plus metadata associated with the entire study, or with its specific parts, such as individual data files. The accompanying metadata includes fields such as title, authors and submission date, making the BioStudy citable. Further structured metadata are optional and can describe how data files were generated, or give biological context (such as tissue sampled). These fields alongside a free‐text study description field provide the content basis for searching across datasets.

BioStudies will ingest data from Europe PMC as well as taking direct submissions. When an article is deposited in Europe PMC and mentions the persistent identifier of a dataset (Accession number or DOI) in the text, or has supplemental data attached to the article, the BioStudies metadata as outlined in Box 1 will be generated automatically and deposited in BioStudies. This will provide a collated view of all the data associated with an article, supporting the Use Cases described above. The accession numbers and data DOIs are extracted by the Europe PMC text‐mining pipeline (Kafkas *et al*, 2013), which currently covers 20 major data resources in the life sciences (ENA, SNPs, PDBe, RefSeq, ClinicalTrials.gov, EudraCT, OMIM, GO, UniProt, Pfam, ArrayExpress, Ensembl, InterPro, BioProject, Proteome Exchange, BioSample, Embd, TreeFam, EGA and data DOIs).

**Box 1: Example of a BioStudies record**

The study S‐EPMC3044716 (http://www.ebi.ac.uk/biostudies/studies/S-EPMC3044716) relates to the *PLoS ONE* paper: “Regulation of the DNA damage response and gene expression by the Dot1L histone methyltransferase and the 53Bp1 tumour suppressor” (FitzGerald *et al*, 2011). The authors have submitted their two transcriptomics datasets to ArrayExpress (Accession MEXP‐2721, E‐MEXP‐2722) and have also referred to the DOT1L locus in Ensembl (ENSGALG00000000843). Some additional information was included with the paper as supplementary material: primer sequences (Table S1 in FitzGerald *et al*, 2011; http://www.ebi.ac.uk/biostudies/files/S-EPMC3044716/pone.0014714.s009.docx) and lists of over‐represented genes grouped by GO term (http://www.ebi.ac.uk/biostudies/files/S-EPMC3044716/pone.0014714.s010.pdf). All this information is aggregated in a single BioStudies record (see figure below). In this simple case, the BioStudies record consists of the title, author names and affiliations from the article, the article abstract, links to the full article in Europe PMC, data referred to in the article narrative in two different resources and the supplemental data files associated with the article. If the article record has information on the funding source or ORCIDs linked to the authors, then these would also be included.

**Box 1 Figure.** A BioStudy aggregating links to Europe PMC, ArrayExpress, Ensembl, and supplementary data files that do not fit into structured repositories.

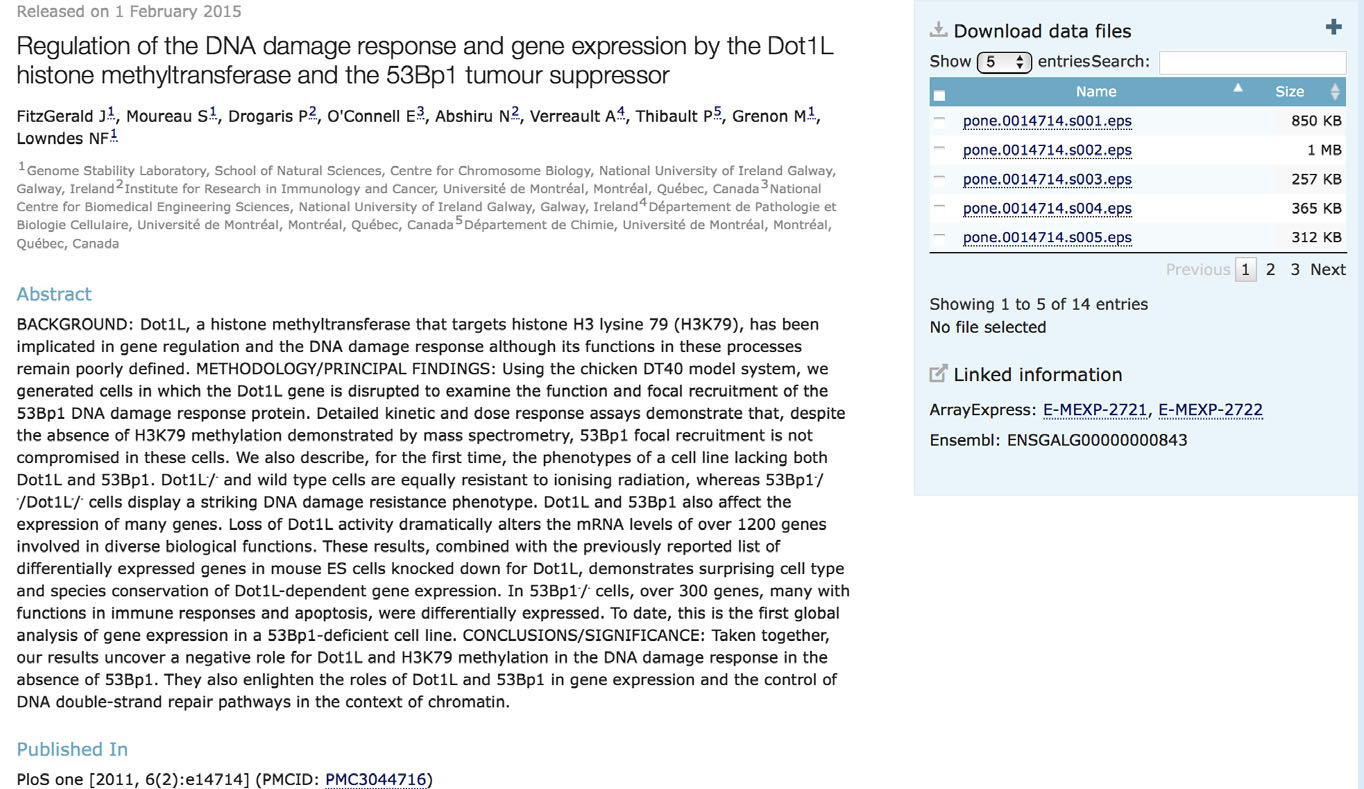

In this example, the author names, affiliations and study description (abstract) have been imported directly from the Europe PMC article, but if this study had been deposited directly, then the submitter can enter this information via the submission form and include other structured information. The study description can be added as free text and can be structured into sections, should that be desirable for more extensive studies. All submissions to BioStudies are assigned a stable accession number at the level of the study.

## Discussion and future work

BioStudies will include study data relating any of the EMBL‐EBI archival databases. Currently, BioStudies accepts submissions from large projects and will accept individual submissions in late 2015. We expect that BioStudies will be capable of storing large files (such as image sets), fitting with other big data community requirements met at the EMBL‐EBI. As an EMBL‐EBI archive, there is a long‐term commitment to supporting BioStudies, for as long as it is useful to the scientific community.

In time, it will be possible to develop automated approaches to content enrichment, for example overlaying ontological terms to provide biology‐sensitive search features. Since the BioStudies implementation is flexible and extensible, research communities or projects will be able to define specific metadata attributes if they wish, supported by easy‐to‐customize submission tools. Also, project‐specific rendering of study data will be supported, allowing us to build project portals within BioStudies. The ability for submitters to edit or extend metadata, or for others to comment, will provide a temporal continuity to the study that is not supported by article publication alone. As credit systems extend from articles to datasets, and even to comments, BioStudies will become a vital part of the infrastructure delivering impact indicators regarding data citation and reuse. In this regard, the use of universal identifier resolution systems will also be explored in order to understand citation dynamics across different workflows in scientific publishing systems.

One of the biggest challenges of enabling reproducible science is to ensure that data can be found and is usable by others. We need to ensure that data resources already meeting this need remain intact, while providing new solutions for new data types. Balancing the management of unstructured datasets, articles and structured data effectively will be critical to maximizing future data‐driven scientific opportunities. Equally, data providers and data infrastructures should not be overburdened with cumbersome or costly processes that do not match the use case. These are the key reasons to develop BioStudies at the EMBL‐EBI, in the context of existing data and literature resources, and building on years of institutional expertise in open data for the life sciences.

We will work with the publisher, submitter and user communities to design the best possible approaches to biological information management, integration and reuse. The flexible infrastructure of BioStudies manages life sciences data of all descriptions and sizes, including raw data from technologies for which no structured archives yet exist. In this way, BioStudies will contribute to effective data management to meet the requirements of life sciences research in future.

## References

[msb156658-bib-0001] Bloom T , Ganley E , Winker M (2014) Data access for the open access literature: PLOS's data policy. PLoS Biol 12: e1001797

[msb156658-bib-0003] Data Citation Synthesis Group (2014) Joint Declaration of Data Citation Principles, Martone M. (ed.). San Diego, CA: FORCE11 https://www.force11.org/datacitation

[msb156658-bib-0004] Editorial Nature Cell Biology (2014) An update on data reporting standards. Nat Cell Biol 16: 385 24784848 10.1038/ncb2964

[msb156658-bib-0005] FitzGerald J , Moureau S , Drogaris P , O'Connell E , Abshiru N , Verreault A , Thibault P , Grenon M , Lowndes NF (2011) Regulation of the DNA damage response and gene expression by the Dot1L histone methyltransferase and the 53Bp1 tumour suppressor. PLoS ONE 6: e14714 21383990 10.1371/journal.pone.0014714 PMC3044716

[msb156658-bib-0006] International Union of Crystallography Commission on Biological Macromolecules (1989) Policy on publication and the deposition of data from crystallographic studies of biological macromolecules. Acta Crystallogr A45: 658

[msb156658-bib-0007] Kafkas S , Kim JH , McEntyre J (2013) Database citation in full text biomedical research articles. PLoS ONE 8: e63184 23734176 10.1371/journal.pone.0063184 PMC3667078

[msb156658-bib-0008] Kahn P , Hazledine D (1988) NAR's new requirement for data submission to the EMBL data library: information for authors. Nucleic Acids Res 16: I–IV 16617480 PMC336623

[msb156658-bib-0009] National Research Council (US) Committee on Responsibilities of Authorship in the Biological Sciences (2003) Sharing Publication‐Related Data and Materials: responsibilities of Authorship in the Life Sciences. Washington, DC: National Academies Press (US) 22649805

[msb156658-bib-0002] Royal Society (2012) Science as an Open Enterprise: Final Report. London, UK: The Royal Society https://royalsociety.org/~/media/policy/projects/sape/2012-06-20-saoe.pdf

